# Psychological symptoms associated with self-reported events of COVID-19 contact, symptoms, or diagnosis: a large community-based survey among adults in Quebec, Canada

**DOI:** 10.17269/s41997-022-00637-5

**Published:** 2022-04-18

**Authors:** Mélissa Généreux, Elsa Landaverde

**Affiliations:** 1 Department of Community Health Sciences, Faculté de Médecine et des Sciences de la Santé, Université de Sherbrooke, 3001 12e Avenue Nord Immeuble X1, Sherbrooke, QC J1H 5N4 Canada; 2 Centre Intégré Universitaire de Santé et de Services Sociaux de l’Estrie - Centre Hospitalier Universitaire de Sherbrooke, Sherbrooke, QC J1G 1B1 Canada

**Keywords:** COVID-19, Major depression episode, Psychological response, Pandemic, COVID-19, dépression majeure probable, réponse psychologique, pandémie

## Abstract

**Objectives:**

Psychological consequences of COVID-19 contact, symptoms, or diagnosis are being increasingly reported. Few studies have examined the psychological effects tied to these events, using an unaffected comparison group. Most did not consider confounding factors like fear and stigma. This study aims to (1) identify individual characteristics associated with COVID-19 contact/symptoms or diagnosis and (2) examine the independent association between COVID-19 contact/symptoms or diagnosis and psychological symptoms.

**Methods:**

From September 2020 to February 2021, 20,327 adults participated in community-based surveys in Quebec. Using repeated cross-sectional online questionnaire, data were collected on probable generalized anxiety disorder (GAD) and major depression episode (MDE), using the GAD-7 and the PHQ-9 scales, respectively. Self-reported events of (1) contact with a case or symptoms of COVID-19, and (2) diagnosis of COVID-19 were examined, along with several sociodemographic and pandemic-related factors.

**Results:**

COVID-19 contact, symptoms, or diagnosis was more frequent in young adults, healthcare or social services workers, adults living with children, and those reporting a greater sense of threat, stigma, financial losses, or daily stress. COVID-19 contact or symptoms and diagnosis were associated with probable MDE relative to the unaffected group (adjusted odds ratio [aOR]: 1.25, 95% CI: 1.12–1.39 and aOR:1.82, 95% CI: 1.48–2.2, respectively). Suicidal thoughts and psychomotor retardation were the symptoms most closely associated with a COVID-19 diagnosis.

**Conclusion:**

Results from this study stress the need for better understanding, recognition, and support for people suffering from psychological symptoms following a COVID-19 diagnosis.

## Résumé

**Objectifs:**

Les conséquences psychologiques d’un contact, de symptômes ou d’un diagnostic de COVID-19 sont de plus en plus signalées. Peu d’études ont examiné les effets psychologiques liés à ces évènements en utilisant des groupes non affectés et n’ont pas tenu compte des facteurs de confusion tels que la peur et la stigmatisation. Cette étude vise à 1) identifier les caractéristiques associées au contact/symptômes ou diagnostic de COVID-19 et 2) examiner l’association entre un contact/symptômes ou diagnostic de COVID-19 et les symptômes psychologiques.

**Méthodes:**

Un total de 20 327 adultes ont participé à une enquête transversale populationnelle au Québec entre septembre 2020 et février 2021. À l’aide d’un questionnaire en ligne, des données ont été recueillies sur l’anxiété généralisée probable (GAD-7) et la dépression majeure probable (PHQ-9). Les évènements autodéclarés de 1) contact avec un cas ou de symptômes de COVID-19 et 2) diagnostics de COVID-19 furent examinés, ainsi que plusieurs facteurs sociodémographiques et liés à la pandémie.

**Résultats:**

Les contacts, symptômes ou diagnostics de COVID-19 étaient plus fréquents chez les jeunes adultes, les travailleurs de la santé, les adultes vivant avec des enfants, et ceux rapportant plus de stigmatisation, pertes financières, stress quotidien ou un plus haut niveau de menace perçue. Des contacts ou symptômes de COVID-19 et un diagnostic de COVID-19 étaient associés à de la dépression majeure probable comparativement au groupe non affecté (RCa : 1,25, IC à 95% : 1,12-1,39 et RCa : 1,82, IC à 95% : 1,48-2,2, respectivement). Les pensées suicidaires et le retard psychomoteur étaient aussi plus étroitement associés à un diagnostic de COVID-19.

**Conclusion:**

Les résultats de cette étude soulignent le besoin d’améliorer nos connaissances et notre soutien pour les personnes souffrant de symptômes psychologiques suite à un diagnostic de COVID-19.

## Introduction

As we now have a more accurate understanding of the acute symptoms of SARS-CoV-2 infection, a vast array of persistent symptoms are being increasingly reported, including various neuropsychiatric symptoms such as difficulty thinking or concentrating (referred to as “brain fog”) or anxiety and depression. Some question whether these persistent symptoms may be related to long COVID, a syndrome corresponding to the persistence of symptoms for several weeks to months in people recovered from COVID-19 (Carfì et al., 2020; Davis et al., 2021; CDC, 2020). However, it is particularly difficult to disentangle these symptoms from the increase in psychological symptoms seen in the general population caused by pandemic-related stressors, including fear, stigma, financial losses, isolation, and quarantine (Généreux et al., 2020; Wang et al., 2020; Kämpfen et al., 2020; Jenkins et al., 2021; Huang & Zhao, 2020; Zürcher et al., 2020). Due to the extensive research on pandemic-related stressors, their relationship has become increasingly understood. In fact, economic concerns, perceived level of threat, and being a victim of stigma emerged as some of the strongest predictors of declining mental health (Kämpfen et al., 2020; Généreux et al., 2021). In addition, a cross-sectional, nationwide study in China found that quarantine measures were associated with higher risk of psychological impacts when adjusted for potential confounders (Wang et al., 2021). Various studies also found gendered impacts of the pandemic on mental health with women more likely to experience negative mental health outcomes (Généreux et al., 2021; Jenkins et al., 2021). On the contrary, the independent relationship between COVID-related events (including contact, symptoms, or diagnosis) and psychological impacts remains unclear.

Recent studies have demonstrated that individuals who have experienced a diagnosis of COVID-19 may potentially be affected by strong psychological effects. An Italian study among 226 COVID survivors observed that 24.3% of patients met the criteria for the diagnosis of a psychiatric disorder (e.g., major depression, anxiety, insomnia), with symptoms remaining after 3 months (Mazza et al., 2021). In the United States, a large study compared the rate of psychiatric diagnosis among cases of COVID-19 and patients affected by other health events. The estimated probability of having been diagnosed with psychiatric illnesses 14 to 90 days after a COVID-19 diagnosis was 18%, significantly higher than for all control health events (Taquet et al., 2021). Severe symptoms of depression post-COVID were also reported elsewhere (Liu et al., 2020; Raman et al., 2021; Mazza et al., 2020). Other studies have observed neurocognitive deficits (e.g., verbal memory deficits, trouble concentrating) in patients having had COVID-19 (Rass et al., 2020; Tomasoni et al., 2021; Ferrucci et al., 2021), some being possibly associated with a psychiatric disorder. Although gender- and age-based results were rarely presented, a study by Mazza et al. (2020) observed that younger and female COVID-19–recovered patients expressed higher levels of negative psychological outcomes.

To date, very few studies have examined the long-term psychological effects of events of COVID-19 contact, symptoms, or diagnosis using unaffected comparison groups, and most did not consider major confounding factors. A better understanding of the relationship between these events and long-term mental health impacts, independent from the influence of other COVID-19-related factors, is key in orienting our collective efforts to fight the global threat posed by COVID-19 (Callard and Perego, 2021; Carfì et al., 2020). The objectives of our study were therefore to:

1. Identify individual characteristics associated with COVID-19 contact/symptoms or diagnosis, and
2. Examine the independent association between COVID-19 contact/symptoms or diagnosis and psychological symptoms.

## Methods

### Design

This survey is part of a two-year international project financed by the Canadian Institutes of Health Research. It was reviewed and approved by the Research Ethics Board of the CIUSSS de l’Estrie – CHUS (HEC ref: 2020-3674). The community-based surveys fall into the first axis of the larger international study, where repeated cross-sectional online questionnaires were completed by adults in eight countries (Généreux et al., 2020; Généreux et al., 2021). In parallel to the international study, a Quebec survey was carried out September 4–14, 2020, November 6–18, 2020, and February 5–16, 2021.

### Recruitment

Recruitment and data collection were undertaken by a professional polling firm. The sample was drawn randomly from Web panels including adults 18 years and older residing in the province of Quebec, Canada. Members of the panels were recruited voluntarily using a variety of strategies (random recruitment, in social media, through campaigns or partners) (refer to Généreux et al., 2020 for more details). Efforts were made to maximize the representativeness by using software generating representative samples of the population and by including hard-to-reach groups through targeted recruitment. Minimum quotas representing 70% of the population distribution were reached during recruitment for age categories, sex, and regions to ensure the best possible representation of the sample. All data were then weighted for sex, age, language, and region of residence according to data from the Statistics Canada 2016 census in order to reach a highly representative sample. The survey was anonymous; therefore, all personal information remained confidential. In case of duplicates (i.e*.*, more than one participation in the survey), only the most recent participation was included in the final sample.

### Measurement

A pre-tested open online questionnaire, available in French and English, was used for data collection. It contained just over 80 closed questions (about 20 min); however, adaptive questioning could have shortened the questionnaire. Each question appeared once the previous question was completed. A nonresponse option was provided for questions concerning sensitive topics. Details about the length, purpose, and consent to the study were provided before the start of the survey.

### Variables

First, probable generalized anxiety disorder (GAD) and major depression episode (MDE) were measured using the GAD-7 and the Patient Health Questionnaire-9 (PHQ-9) scales, respectively (Swinson, 2006; Levis et al., 2019). These questionnaires, based on DSM-IV diagnostic criteria, are designed for use by health professionals but are also regularly used in population-based studies. The GAD-7 (7 items) has a composite score ranging between 0 and 21 while the PHQ-9 (9 items) score ranges from 0 to 27 (Swinson, 2006; Levis et al., 2019). For both scales, combined sensitivity and specificity have been shown to be maximized at a cutoff score of 10 or above. A score ≥ 10 indicates moderate or severe symptoms, while a score ≥ 15 indicates severe symptoms (Swinson, 2006; Levis et al., 2019). Specific symptoms of MDE were also assessed post hoc based on the items of the PHQ-9 scale in order to evaluate the prevalence of each symptom individually. The same analyses were performed, but for each of the 9 items rather than for the overall score of 10 or more on the PHQ-9. The symptoms were considered “frequent” if they were present at least half of days over the last 2 weeks.

The main exposure variable was based on self-reported events of COVID-19 contact, symptoms, or diagnosis. Participants were asked which type of COVID-19 experience they had faced since the start of the pandemic. These experiences were classified as (1) no direct COVID-19 experience, (2) having experienced self-isolation or quarantine due to a contact with a case or symptoms of COVID-19 (without diagnosis), or (3) having been diagnosed by a doctor with COVID-19 or having experienced self-isolation or quarantine due to a diagnosis of COVID-19. The first group was considered as a comparison (unaffected) group.

### Covariables

Several factors previously identified as positively or negatively influencing the psychological response to the pandemic were examined following the results of the larger international study into which this study falls (Généreux et al., 2021). In light of the results of this study, factors were chosen to act as confounding variables to observe the independent relationship of self-reported events of a COVID-19 contact, symptoms, or diagnosis. These include the phases of data collection (September 2020, November 2020, or February 2021), having been diagnosed with a high-risk medical condition (heart disease, chronic bronchitis, emphysema, diabetes, immunosuppression), daily stress perceived as high (participants having answered “rather stressful” or “extremely stressful” to the question: thinking about the level of stress in your life, would you say that most of your days are…), threat posed by the coronavirus for oneself and/or family perceived as high (participants having answered “high” or “very high” to the question: what level of threat do you think the coronavirus poses to each of the following?), and being a victim of stigma and having experienced financial losses due to the pandemic (participants having answered “yes” to the question: Due to the coronavirus, have you experienced the following disruptions? a) Victim of stigma or discrimination, b) financial losses). Sociodemographic characteristics were also examined, including sex, age, language spoken at home, educational attainment, children at home, and being a healthcare or social services worker.

### Data analysis

The data from completed questionnaires were analyzed using the SPSS statistics version 26. Chi^2^ tests were used to compare the profile of individuals on the basis of having reported a COVID-19 contact/symptoms or a COVID-19 diagnosis, and to examine bivariate relationships between COVID-19 contact/symptoms or a COVID-19 diagnosis and psychological symptoms (overall, and according to gender and age). Multivariate logistic regression was also performed. Sensitivity analyses using cases that were isolated due to a diagnosis of COVID-19 during wave 1 (March–July 2020) only allowed exploring whether the psychological effects persisted over time (i.e., 1 to 11 months after the COVID-19 experience).

## Results

### Participants and their characteristics

The final sample totalled 20,327 adults (September 2020: 3978; November 2020: 5836; February 2021: 10,513). Among the adults surveyed, 599 adults (3%) had reported being diagnosed with COVID-19 and 3008 (15%) had reported requiring quarantine or self-isolation due to a contact with a case or symptoms of COVID-19 (without diagnosis), for a total of 18% of the sample having had a self-reported event of COVID-19 contact, symptoms, or diagnosis. Of the 599 self-reported cases of COVID-19, 244 were acquired during the first wave.

Table 1 details the sociodemographic characteristics of the participants as well as the various pandemic-related stressors they had been exposed to, according to the three different groups. Any self-reported event of COVID-19, whether by contact, symptoms, or diagnosis, was more frequent among young adults, healthcare, or social services workers, and to a lesser extent among adults living with children. A crude association (*p*≤0.05) was also observed between reporting either a COVID-19 contact, symptoms, or diagnosis and reporting a greater sense of threat, stigma, financial losses, or daily stress.

**Table 1:** Distribution of self-reported events of COVID-19 contact, symptoms, or diagnosis according to various sociodemographic and pandemic-related characteristics (*n*=20,327 adults in Quebec; September 2020 to February 2021)

	No COVID-19–related event	Contact or symptoms	Diagnosis
	*n* (%)	*n* (%)	*n* (%)
Wave of data collection			
September 2020	3117 (85.0%)*	447 (12.2%)*	101 (2.8%)*
November 2020	4762 (83.0%)*	841 (14.7%)*	133 (2.3%)*
February 2021	8632 (80.5%)*	1720 (16.0%)*	365 (3.4%)*
Gender			
Men	7619 (82.2%)*	1296 (14.0%)*	357 (3.9%)*
Women	8838 (82.1%)*	1695 (15.7%)*	238 (2.2%)*
Age			
18–34 years	3782 (71.0%)*	1245 (23.4%)*	297 (5.6%)*
35–49 years	3702 (77.0%)*	956 (19.9%)*	149 (3.1%)*
50–64 years	4726 (87.5%)*	588 (10.9%)*	89 (1.6%)*
65 years and over	4301 (93.8%)*	210 (4.8%)*	64 (1.4%)*
Education			
High school or less	4086 (85.0%)*	593 (12.3%)*	130 (2.7%)*
College	5342 (81.0%)*	1048 (15.9%)*	204 (3.1%)*
University	6957 (81.1%)*	1361 (15.9%)*	260 (3.0%)*
Language			
English	2537 (79.6%)*	527 (16.5%)*	123 (3.9%)*
Other	13,973 (82.5%)*	2480 (14.6%)*	476 (2.8%)*
Children at home			
Yes	12,488 (84.2%)*	1907 (12.9%)*	429 (2.9%)*
No	4022 (76.0%)*	1100 (20.8%)*	170 (3.2%)*
Healthcare or social services worker			
Yes	1114 (69.8%)*	345 (21.6%)*	137 (8.6%)*
No	15,396 (83.1%)*	2663 (14.4%)*	462 (2.5%)*
High-risk medical conditions			
Yes	5726 (84.9%)*	782 (11.6%)*	239 (3.5%)*
No	10,272 (80.5%)*	2168 (17.0%)*	323 (2.5%)*
Threat perceived as high			
Yes	5730 (79.4%)*	1225 (17.0%)*	259 (3.6%)*
No	10,378 (83.3%)*	1755 (14.1%)*	329 (2.6%)*
Being a victim of stigma			
Yes	899 (65.8%)*	309 (22.6%)*	159 (11.6%)*
No	15,103 (83.1%)*	2626 (14.6%)*	432 (2.4%)*
Financial losses			
Yes	2817 (75.8%)*	749 (20.2%)*	148 (4.0%)*
No	13,394 (83.3%)*	2234 (13.9%)*	447 (2.8%)*
High level of stress			
Yes	3792 (73.6%)*	1159 (22.5%)*	199 (3.9%)*
No	12,543 (84.8%)*	1845 (12.5%)*	396 (2.7%)*
Total	16,510 (82.1%)	3008 (15.0%)	599 (3.0%)

*The differences between each type of self-reported event of COVID-19 per subset of sociodemographic and pandemic-related variables are significant at an alpha threshold of 0.005 on the Chi^2^ test

### Psychological symptoms

A higher prevalence of recent psychological symptoms was observed in participants who had reported COVID-19 contact, symptoms, or diagnosis compared to those reporting no COVID-19–related events (Table 2). COVID-19 diagnosis appeared to be associated with an increased risk of probable MDE (38.1%), in comparison to the other comparison groups (no COVID-19–related event: 16.9%, contact or symptoms: 29.0%). By limiting the analysis to cases diagnosed during the first wave of the pandemic, the prevalence of probable MDE remained high (37.7%).

**Table 2:** Crude and adjusted associations between probable major depressive disorders and self-reported events of COVID-19 contact, symptoms, or diagnosis (*n*=20,327 adults in Quebec; September 2020 to February 2021)

Self-reported events of COVID-19	Probable major depressive episode %	Crude odds ratio		Adjusted* odds ratio	
		OR	[CI 95%]	OR	[CI 95%]
No COVID-19–related event (*n*=16,510)	2791 (16.9%)	**1**	**Reference**	**1**	**Reference**
Contact or symptoms (*n*=3008)	871 (29.0%)	**2.00**	**[1.83; 2.19]**	**1.25**	**[1.12; 1.39]**
Diagnosis (*n*=599)	228 (38.1%)	**3.02**	**[2.54; 3.57]**	**1.82**	**[1.48; 2.24]**
Gender strata					
Men					
No COVID-19–related event (*n*=7539)	1122 (14.7%)	**1**	**Reference**	**1**	**Reference**
Contact or symptoms (*n*=1286)	346 (26.7%)	**2.11**	**[1.84; 2.42]**	**1.37**	**[1.16; 1.62]**
Diagnosis (*n*=348)	166 (46.6%)	**5.05**	**[4.06; 6.27]**	**2.49**	**[1.89; 3.27]**
Women					
No experience (*n*=8707)	1646 (18.6%)	**1**	**Reference**	**1**	**Reference**
Contact or symptoms (*n*=1678)	516 (30.4%)	**1.91**	**[1.70; 2.15]**	**1.18**	**[1.03; 1.35]**
Diagnosis (*n*=237)	62 (26.1%)	**1.53**	**[1.14; 2.05]**	**1.10**	**[0.78; 1,54]**
Age strata					
18–34 years					
No COVID-19–related event (*n*=3699)	1074 (28.4%)	**1**	**Reference**	**1**	**Reference**
Contact or symptoms (*n*=1229)	434 (34.9%)	**1.35**	**[1.18; 1.54]**	**1.16**	**[1.00; 1.36]**
Diagnosis (*n*=289)	153 (51.5%)	**2.66**	**[2.10; 3.38]**	**1.85**	**[1.39; 2.47]**
35–49 years					
No COVID-19–related event (*n*=3653)	777 (21.0%)	**1**	**Reference**	**1**	**Reference**
Contact or symptoms (*n*=948)	301 (31.5%)	**1.73**	**[1.48; 2.03]**	**1.54**	**[1.28; 1.85]**
Diagnosis (*n*=146)	56 (37.6%)	**2.29**	**[1.63; 3.22]**	**2.00**	**[1.34; 2.99]**
50–64 years					
No COVID-19–related event (*n*=4677)	607 (12.8%)	**1**	**Reference**	**1**	**Reference**
Contact or symptoms (*n*=584)	113 (19.2%)	**1.61**	**[1.29; 2.01]**	**1.12**	**[0.86; 1.44]**
Diagnosis (*n*=88)	14 (15.7%)	**1.23**	**[0.68; 2.20]**	**1.06**	**[0.55; 2.03]**
65 years and older					
No COVID-19–related event (*n*=4261)	332 (7.7%) (NS)	**1**	**Reference**	**1**	**Reference**
Contact or symptoms (*n*=218)	23 (10.5%) (NS)	**1.39**	**[0.89; 2.17]**	**1.03**	**[0.63; 1.70]**
Diagnosis (*n*=64)	5 (7.8%) (NS)	**1.05**	**[0.42; 2.59]**	**0.82**	**[0.31; 2.21]**
Sensitivity analyses					
COVID-19 diagnosis during wave 1 only (*n*=244)	92 (37.7%)	**2.54**	**[1.96; 3.30]**	**1.72**	**[1.26; 2.35]**

*Adjusted for wave of data collection, sex, age, education, language, children at home, healthcare or social services worker status, high-risk medical conditions, threat perceived for oneself or the family, stigma and financial losses due to the pandemic, and level of daily stress

Findings from bivariate and multivariate analyses shown in Table 2 suggest that the crude relationship observed between self-reported events of a COVID-19 contact, symptoms, or diagnosis and probable MDE was weakened but remained significant after adjusting for various socio-demographic and pandemic-related characteristics. The likelihood of moderate or severe symptoms of MDE was increased by 82% in people who have reported a COVID-19 diagnosis (adjusted odds ratio [aOR] 1.82; 95% CI 1.48–2.24) and by 25% in individuals having reported requiring isolation due to contact or symptoms (aOR 1.25; 95% CI 1.12–1.39), compared to individuals reporting no COVID-19-related events. The two ORs and their respective 95% CIs do not overlap, suggesting a stronger effect of the diagnosis of COVID-19 versus the contact with a case or symptoms of COVID-19 (without diagnosis). The strength of this association weakened slightly when observing the cases diagnosed in the first wave of the pandemic but the association remained significant (aOR 1.72; 95% CI 1.26–2.35). As depicted in Table 2, probable MDE was clearly increased in men having reported a diagnosis of COVID-19 (46.6%) but not in women among whom level of probable depression was similar regardless of the type of COVID-19 event (contact, symptoms, or diagnosis). In fact, for men, the multivariate analysis shows a significant association between MDE and isolation due to COVID-19 contact or symptoms (aOR 1.37; 95% CI 1.16–1.62) and an even stronger association in men who had reported a COVID-19 diagnosis (aOR 2.49; 95% CI 1.89–3.27). A significant association was found in women who had reported COVID-19 contact or symptoms but not in those who had reported a COVID-19 diagnosis. The association between MDE and isolation due to diagnosis of COVID-19 was, however, only noted in younger adults. Young people reporting a diagnosis of COVID-19 showed higher levels of probable MDE (18–34 years 51.5%, 35–49 years 37.6%), compared to both their counterparts in the same age group (without diagnosis) and to older people with a diagnosis. The likelihood of moderate or severe symptoms of MDE was increased by 85% in individuals aged 18–34 years who have reported a COVID-19 diagnosis (aOR 1.85; 95% CI 1.39–2.47) and by 100% in individuals aged 35–49 years who have reported a COVID-19 diagnosis (aOR 2.00; 95% CI 1.34–2.99), compared to individuals reporting no COVID-19–related events. No significant association was noted for older age groups.

In contrast, individuals who had been in contact with a case or who had symptoms of COVID-19 (without diagnosis) presented with levels of probable GAD that were comparable to those of individuals who had reported a diagnosis of the disease (as seen in Table 3). When observing the population sample as a whole, the association between contact, symptoms, or diagnosis of COVID-19 and GAD remained significant after adjusting for various socio-demographic and pandemic-related characteristics. However, there was no significant difference between the two types of events, as the two confidence intervals overlapped. In addition, when limiting the analysis to cases diagnosed during the first wave of the pandemic, the association between diagnosis of COVID-19 and GAD was no longer significant (aOR 0.92; 95% CI 0.63–1.34), suggesting that symptoms of GAD following a COVID-19 diagnosis resolve more quickly. When observed by gender and age, the impacts of COVID-19 events on GAD were found to be insignificant for most groups, as depicted in Table 3.

**Table 3:** Crude and adjusted associations between generalized anxiety disorder and self-reported events of COVID-19 contact, symptoms or diagnosis (*n*=20,327 adults in Quebec; September 2020 to February 2021)

Self-reported events of COVID-19	Probable generalized anxiety disorder %	Crude odds ratio		Adjusted* odds ratio	
		OR	[CI 95%]	OR	[CI 95%]
No COVID-19–related event (*n*=16,510)	2242 (13.6%)	**1**	**Reference**	**1**	**Reference**
Contact or symptoms (*n*=3008)	724 (24.1%)	**2.02**	**[1.84; 2.22]**	**1.23**	**[1.10; 1.38]**
Diagnosis (*n*=599)	153 (25.5%)	**2.18**	**[1.81; 2.63]**	**1.32**	**[1.04; 1.67]**
Gender strata					
Men					
No COVID-19–related event (*n*=7539)	858 (11.3%)	**1**	**Reference**	**1**	**Reference**
Contact or symptoms (*n*=1286)	267 (20.6%)	**2.04**	**[1.76; 2.38]**	**1.32**	**[1.09; 1.58]**
Diagnosis (*n*=348)	118 (33.1%)	**3.92**	**[3.11; 4.94]**	**1.97**	**[1.46; 2.67]**
Women					
No experience (*n*=8707)	1360 (15.4%)	**1**	**Reference**	**1**	**Reference**
Contact or symptoms (*n*=1678)	447 (26.4%)	**1.97**	**[1.74; 2.23]**	**1.20**	**[1.03; 2.03]**
Diagnosis (*n*=237)	34 (14.3%)	**0.93**	**[0.64; 1.34]**	**0.64**	**[0.42; 0.97]**
Age strata					
18–34 years					
No COVID-19–related event (*n*=3699)	881 (23.3%)	**1**	**Reference**	**1**	**Reference**
Contact or symptoms (*n*=1229)	370 (29.7%)	**1.39**	**[1.21; 1.61]**	**1.23**	**[1.03; 1.46]**
Diagnosis (*n*=289)	101 (34.0%)	**1.69**	**[1.31; 2.17]**	**1.26**	**[0.91; 1.73]**
35–49 years					
No COVID-19–related event (*n*=3653)	624 (16.9%)	**1**	**Reference**	**1**	**Reference**
Contact or symptoms (*n*=948)	223 (23.2%)	**1.50**	**[1.26; 1.78]**	**1.13**	**[0.92; 1.40]**
Diagnosis (*n*=146)	34 (22.8%)	**1.48**	**[1.00; 2.19]**	**1.22**	**[0.76; 1.95]**
50–64 years					
No COVID-19–related event (*n*=4677)	483 (10.2%)	**1**	**Reference**	**1**	**Reference**
Contact or symptoms (*n*=584)	101 (17.2%)	**1.82**	**[1.44; 2.29]**	**1.17**	**[0.89; 1.55]**
Diagnosis (*n*=88)	11 (12.4%)	**1.25**	**[0.66; 2.36]**	**1.15**	**[0.57; 2.34]**
65 years and older					
No COVID-19–related event (*n*=4261)	253 (5.9%)	**1**	**Reference**	**1**	**Reference**
Contact or symptoms (*n*=218)	31 (14.1%)	**2.61**	**[1.75; 3.90]**	**2.03**	**[1.26; 3.29]**
Diagnosis (*n*=64)	7 (10.8%)	**1.85**	**[0.82; 4.17]**	**1.52**	**[0.58; 3.95]**
Sensitivity analyses					
COVID-19 diagnosis during wave 1 only (*n*=244)	51 (20.9%)	**1.44**	**[1.06; 1.97]**	**0.92**	**[0.63; 1.34]**

*Adjusted for wave of data collection, sex, age, education, language, children at home, healthcare or social services worker status, high-risk medical conditions, threat perceived for oneself or the family, stigma and financial losses due to the pandemic, and level of daily stress

Since a significant relationship was found between events of COVID-19 contact, symptoms, or diagnosis and symptoms of MDE, the prevalence of each depressive symptom, corresponding to the 9 items of the PHQ-9 scale, was examined based on the different types of COVID-19–related events (Table 4). These analyses indicated that some depressive symptoms were more closely related to a COVID-19 diagnosis, including disturbed appetite, guilt, concentration difficulties, psychomotor slowdown, and suicidal thoughts. In fact, no fewer than 17.2% of people recovered from COVID-19 reported having suicidal thoughts (i.e., thoughts that they would be better off dead or hurting themselves in some way) at least every other day in the past 2 weeks, a proportion higher than in the “contact/symptoms” (6.8%) and the “no experience” groups (4.3%). Psychomotor slowdown was also significantly increased in individuals having reported a COVID-19 diagnosis as compared to the other two groups. Furthermore, proportions of suicidal thoughts and psychomotor slowdown remained elevated even among people who reported a COVID-19 diagnosis in the first wave of the pandemic.

**Table 4:** Frequent symptoms of major depression episode (present at least half of days over the last 2 weeks) according to self-reported events of COVID-19 contact, symptoms, or diagnosis (*n*=20,327 adults in Quebec; September 2020 to February 2021)

	Sadness *n* (%)	Loss of interest *n* (%)	Fatigue *n* (%)	Disturbed sleep *n* (%)	Disturbed appetite *n* (%)	Guilt *n* (%)	Concentration difficulties *n* (%)	Psychomotor slowdown *n* (%)	Suicidal thoughts *n* (%)
Main analyses									
No COVID-19–related event (*n*=16,510)	2095 (12.7%)_a_	2128 (12.9%)_a_	3370 (20.4%)_a_	3145 (18.9%)_a_	2115 (12.8%)_a_	1879 (11.4%)_a_	1661 (10.1%)_a_	796 (4.8%)_a_	706 (4.3%)_a_
Contact or symptoms (*n*=3008)	643 (21.4%)_b_	594 (19.7%)_b_	1040 (34.6%)_b_	924 (30.7%)_b_	651 (21.6%)_b_	608 (20.2%)_b_	521 (17.3%)_b_	233 (7.7%)_b_	204 (6.8%)_b_
Diagnosis (*n*=599)	143 (23.9%)_b_	128 (21.4%)_b_	211 (35.2%)_b_	172 (28.7%)_b_	169 (28.2%)_c_	153 (25.5%)_c_	159 (26.5%)_c_	133 (22.2%)_c_	103 (17.2%)_c_
Sensitivity analyses									
COVID-19 diagnosis during wave 1 only (*n*=244)	58 (23.8%)	54 (22.1%)	84 (34.3%)	72 (29.5%)	68 (27.9%)	62 (25.4%)	53 (21.7%)	53 (21.7%)	36 (14.7%)
Total	2881 (14.3%)	2850 (14.2%)	4621 (23.0%)	4221 (21.0%)	2935 (14.6%)	2640 (13.1%)	2341 (11.6%)	1162 (5.8%)	1013 (5.0%)

Note: *Z*-tests were performed for each symptom of major depression episode according to the COVID-19 events. Subsets of COVID-19 events with the same subscript letter have proportions that do not differ significantly from each other at the 0.05 level

## Discussion

The results of this study contribute to enhancing our current understanding of the psychological symptoms observed in individuals who have reported COVID-19 contact, symptoms, or diagnosis. First, it points to the fact that anxiety may be explained by the psychological stress caused by factors such as isolation and fear tied to events of COVID-19 contact, symptoms, or diagnosis, as both individuals who either had been in contact with a case or who had symptoms (without diagnosis) and those having received a diagnosis displayed similar anxiety levels. In contrast, the report of recent major depression symptoms was far more frequent among people having reported a COVID-19 diagnosis in the past year, with as many as 4 out of 10 reporting moderate or severe depressive symptoms. Perhaps even more worrisome is the fact that suicidal thoughts were among the symptoms most closely associated with the experience of a previous infection, with prevalence four times higher in COVID-19 patients relative to the general population.

Our findings are consistent with those established in a previous study. Personal experiences related to the COVID-19 pandemic have been associated with clinically significant higher levels of stress, anxiety, depression, and functional impairment. An online survey among 565 American adult respondents evaluated participants’ experiences during the coronavirus pandemic. Participants who reported having received a diagnosis of COVID-19 had the highest odds of a probable anxiety/depression diagnosis (Gallagher et al., 2020). When observing moderate to severe cases of COVID-19 2 to 3 months after the disease onset, a higher proportion of COVID-19 patients reported symptoms of depression (39% versus 17%) and anxiety (35% versus 10%) compared to uninfected controls (Raman et al., 2021). This study did not, however, use people who have reported COVID-19 contact or symptoms (without diagnosis) as a comparison group to disentangle the effects of the infection from other stressors. Finally, another study including 135 individuals diagnosed with COVID-19 who agreed to a neurological follow-up 3 months after disease onset between April 2020 and September 2020, found that cognitive deficits were present in 23% of patients (Rass et al., 2020). These results were found to be similar to the proportions of participants diagnosed with COVID-19 in our study reporting psychomotor slowdown or trouble concentrating.

Interestingly, the relationship between a COVID-19 diagnosis and probable MDE was only seen in men and young adults. Few studies have reported results according to gender and age. In Italy, one month post-COVID, younger recovered patients expressed higher levels of depression (Mazza et al., 2020). However, this study reported that females suffered more in all examined psychopathological dimensions (Mazza et al., 2020). These results may stem from the fact that women are generally more at risk of anxiety or depression (Généreux et al., 2020; Wang et al., 2020), potentially skewing results if not taken into consideration. Given the differences noted according to gender and age, future studies should focus on gender- and age-based analyses of psychological impacts of COVID-19 diagnosis in order to better understand this response mechanism.

As seen in the sensitivity analyses, depressive symptoms reported in the last 2 weeks before the date of collection (September 2020, November 2020, and February 2021) were found to be just as frequent in people infected during the first wave of the pandemic as in those infected more recently (both 38%), suggesting that the psychological effects of a COVID-19 diagnosis may last several months following the event. This prevalence may also be higher than originally expected as it has been estimated that 10–20% of people infected with SARS-CoV-2 exhibit symptoms associated with long COVID, which can include psychological symptoms, within weeks or months of recovery (Sudre et al., 2020; Office for National Statistics, 2020).

Classic psychological factors like fear, stigma, and financial losses only seem to explain a part of the association existing between COVID-19 experience and psychological symptoms. This suggests that a pathophysiological mechanism may be involved. It is hypothesized that the results obtained in this study may be associated with the syndrome of long COVID (Carfì et al., 2020; Davis et al., 2021). Due to the cross-sectional nature of this study, the evolution of the observed symptoms over time cannot be assessed; therefore, longitudinal studies are required to confirm this hypothesis. Although this study did not intend to explore the biological pathways involved in the psychological impacts noted, a few possible explanations have been identified in the literature. The severity of depressive symptomatology may indeed be predicted by systemic inflammation during acute infection or by its pattern of change over time (Mazza et al., 2021). It may also be correlated with the severity of the COVID-19 episode (Liu et al., 2020) or with breathlessness (Raman et al., 2021).

This study had several methodological strengths. First, it utilized a large and representative sample of adults including nearly 600 cases of COVID-19. It also places an emphasis on self-reported psychiatric symptoms, which is crucial as clinical observations underestimate the situation due to a reduction in non-urgent medical care utilization in times of pandemic (Direction régionale de santé publique de Montréal, 2020). Contrary to other studies, two comparison groups were considered, with one including individuals who have reported requiring isolation due to a contact with a case or due to having symptoms similar to those observed with COVID-19 (without diagnosis). Finally, a wide range of confounding factors were taken into consideration. This study also had a number of limitations. Its design (i.e., repeated cross-sectional surveys) prevents the ability to infer causality between COVID diagnosis and psychological symptoms. Furthermore, the self-reported nature of the COVID-19 diagnosis may have led to non-differential misclassification bias. The severity of COVID-19 was not examined, nor the precise date of the diagnosis; therefore, no conclusion could be drawn on their impacts on psychological symptoms.

## Conclusion

In accordance with the call recently launched by the World Health Organization (Wise, 2021), results from this study stress the need for better understanding, recognition, and support for people suffering from persistent psychological effects following a COVID-19 diagnosis. Rehabilitation efforts could be enhanced through the establishment of a network of post-COVID clinics composed of multidisciplinary teams able to detect and provide care to people displaying vague symptoms which could pass unnoticed otherwise. Ultimately, these disturbing findings may also encourage those who did not have COVID-19 yet to maintain their efforts in the fight against COVID-19 despite an obvious collective fatigue.

## Contributions to knowledge

What does this study add to existing knowledge?

- Many studies have presented the effects of the pandemic on psychological health in the general population but few of these present the increased risk among individuals who have reported being in contact with a case, experiencing symptoms of COVID-19, or receiving a diagnosis of COVID-19.
- Unlike other similar studies, a comparison group was used to compare the indication of negative psychological outcomes between the different types of COVID-19 events (contact, symptoms, or diagnosis) and the general population. In addition, this study assessed these impacts while taking many potential confounding factors into consideration such as isolation, financial insecurities, and sociodemographic variables.

What are the key implications for public health interventions, practice or policy?

- The results obtained in this study may encourage the need for recognition and support for people suffering from persistent psychological effects following an experience of COVID-19 diagnosis.
- The results of the study may also urge further research to be conducted in order to increase the comprehension of these effects, especially through the use of longitudinal studies, which can allow a more thorough understanding of the evolution of the persistent psychological symptoms noted in this study.

## Data Availability

Not applicable
